# The Story of the Insane from Year to Year

**Published:** 1897-05-08

**Authors:** 


### THE STORY OF THE INSANE FROM YEAR TO YEAR.

Ninth Series.

### EXETER BOROUGH ASYLUM.

Here the numbers varied but little, there being 366 at the beginning of the year and 364 at the close. There were 95 admissions, 7 readmissions, 39 recoveries, 6 discharged relieved, and 29 deaths. The recoveries stand at 45·8 per cent. of the admissions, and the deaths at 7·6 per cent. on the average number resident. The former is above the average of public asylums, and the latter below it. Cerebral and spinal diseases account for 12 of the deaths, and consumption for 3. Turning to table X., which shows the causes of insanity, we find that 16 of the year's admissions were driven mad by drink and 30 by hereditary tendency, and so the same monotonous tale has to be told every year in every asylum in the country. Dr. Rutherford's report is a very short one, and does not contain anything worthy of extraction. We are pleased to notice, however, that several of the staff have obtained the Nursing Certificate of the Medico-Psychological Association. The committee say that "the high praise afforded by them in past years to Dr. Rutherford for the great ability displayed by him in the management of the institution must be continued." The Commissioners' report is not given, and we could not find the usual table showing the weekly cost; but on referring to the Blue Book we find it to be a fraction under 10s. per head.

### WILTS COUNTY ASYLUM, DEVIZES.

On January 1st the numbers were 719, and on the last day of December 744. There were 130 admissions, 30 re-admissions, 49 discharged recovered, 5 discharged relieved, and 68 deaths. The recoveries stand at 30·6 per cent. on the admissions, and the deaths at 9·1 per cent. on the average number resident. Of the deaths 24 were due to cerebral and spinal diseases, and 12 to consumption. Moral causes, such as domestic trouble, religion, and love, account for 21 of the year's admissions, intemperance in drink for 12, hereditary influence and congenital defect for 30. The asylum has accommodation for 725 patients, so that there was considerable overcrowding, but new wards are being built to relieve the pressure on the space. Mr. Bowes shows that during the last twenty years the patients have increased from 878 to 966, and during the same time the numbers of the insane in Wiltshire either living with their friends or in workhouses have decreased respectively from 19 and 24 per cent. to 14 and 11 per cent. We are glad to record Mr. Bowes' opinion that the gross number of Wiltshire lunatics is not on the increase. When speaking of treatment, the wet-sheet pack is correctly described as a humane, rational, and beneficial source of treatment, and we are sorry to hear that such restrictions have been placed upon its use as to cause its abandonment. We congratulate Attendant Alfred Bendall on receiving his pension. The committee refer to the admirable manner in which the medical superintendent has managed the asylum. The weekly rate of maintenance is 8s. 7d. per head.

### KILKENNY DISTRICT ASYLUM.

The limits of accommodation are given at 295; but the asylum contained 346 patients at the beginning of the year and 355 at the end of it. It would be interesting to know who is to blame for this overcrowding. There were 41 admissions, 5 readmissions, 16 recoveries, and 21 deaths. The recoveries stand at 35 per cent. and the deaths at 4 per cent., both reckoned in the usual way. The recovery-rate is a little below the average, and the death-rate very much below the average. Only 5 of the deaths were caused by cerebral diseases, but consumption caused death in 10 cases. Drink is the assigned cause of insanity in 2 of the admissions, and heredity in 17; but as 46 are put down as of unknown origin, manifestly the table is not of much use. Additions are being made to the asylum; but Dr. Myles thinks that patients will be transferred from the workhouses "and will take up all the available accommodation provided by the new additions." As this is a prediction only too likely to be fulfilled, it would surely be well to set about further additions while there is breathing time, and not wait until over-crowding again takes place. Classes are formed by the assistant medical officer for training the attendants preparatory for the examination for certificates of proficiency, and we echo Dr. Myles' hope that those who pass will obtain some recognition of their success from the board of management. Rewards may not be the highest form of inducement to work, but they help in these degenerate days. The average annual cost per head is £18 17s.

### NEWCASTLE-ON-TYNE ASYLUM.

There were 470 patients in residence on January 1st, and 493 on December 31st. The admissions were 118; re-admissions, 16; recoveries, 40; and deaths, 56. Calculated on the admissions, the recoveries stand at 29·8 per cent., and the deaths at 11·5 on the average number resident. Of the deaths, 31 were caused by cerebral and spinal diseases, 11 by consumption, and 4 by pneumonia. Intemperance in drink accounts for 41 of the admissions, and hereditary influence for 38. The asylum is full, but extensive additions are being built; and in the meantime 30 patients have been sent to another asylum, where 14s. a week each is being paid for them. This sum is 5s. 8d. higher than the cost at Newcastle, so that the borough ratepayer has to find £440 a year more for the pauper lunatics than was necessary if the additions had been commenced earlier. There was a slight outbreak of scarlet fever during the year, and it is supposed the infection reached the asylum through the friends of a patient visiting him. The visitors say that Dr. Callcott and the other officers have discharged their duties to their entire satisfaction.

### FIFE AND KINROSS ASYLUM.

The numbers increased from 441 to 474. There were 104 admissions, 31 readmissions, 48 discharged recovered, 22 discharged relieved, and 31 deaths. The percentage of recoveries on admissions is 37·28, and that of the deaths on the average number resident is 9·27. Of the deaths, 12 were caused by brain diseases and 8 by consumption. Intemperance in drink accounts for 23 of the year's admissions, hereditary tendency for 52, and various moral causes for 25. In reading these asylum reports we are always struck with the parts played by alcohol and heredity in the causation of insanity, and we are equally struck with the apparent neglect of all remedies likely to lessen these evils. In a higher and better state of civilisation 75 out of 135 cases admitted to the Fife and Kinross Asylum would not have existed at all, and instead of the tendency being to make larger and larger calls on the asylum accommodation, as Dr. Turnbull tells us is the fact, the asylum would be much too large could these causes be wiped away. In the male sick-room Dr. Turnbull has replaced male attendants by female attendants, and the change has been thoroughly successful. Male attendants are apparently still employed at night in the sick-room, but we do not hear how the bathing arrangements are carried out. The plan of having nurses in the male wards has not been too successful in English asylums, and we shall watch Dr. Turnbull's experiment with much interest. The average weekly cost is 8s. 10d. per head.

